# Metals in Biotechnology: Cr‐Driven Stereoselective Reduction of Conjugated C=C Double Bonds

**DOI:** 10.1002/cbic.201900685

**Published:** 2019-12-19

**Authors:** Marine C. R. Rauch, Yann Gallou, Léna Delorme, Caroline E. Paul, Isabel W. C. E. Arends, Frank Hollmann

**Affiliations:** ^1^ Department of Biotechnology Delft University of Technology Van der Maasweg 9 2629HZ Delft The Netherlands; ^2^ Faculty of Science Utrecht University Budapestlaan 6 3584CD Utrecht The Netherlands

**Keywords:** conjugated double bonds, direct regeneration, metals, old yellow enzymes, stereoselective reduction

## Abstract

Elemental metals are shown to be suitable sacrificial electron donors to drive the stereoselective reduction of conjugated C=C double bonds using Old Yellow Enzymes as catalysts. Both direct electron transfer from the metal to the enzyme as well as mediated electron transfer is feasible, although the latter excels by higher reaction rates. The general applicability of this new chemoenzymatic reduction method is demonstrated, and current limitations are outlined.

## Graphical abstract

**OYE, OYE! Heavy metal and biocatalysis**: Elemental metals are shown to be suitable sacrificial electron donors to drive the stereoselective reduction of conjugated C=C double bonds using Old Yellow Enzymes (OYEs) as catalysts. In this contribution we demonstrate that chemo‐ and enantioselective reductions catalyzed by OYEs can be driven by elemental metals as stoichiometric reductants.

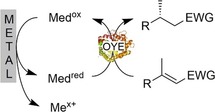

In recent years, so‐called Old Yellow Enzymes (OYEs) have attracted significant attention as catalysts for the stereoselective reduction of conjugated C=C double bonds.^1^ To maintain their catalytic cycle, OYEs depend on a continuous supply with reducing equivalents, which classically originate from the natural nicotinamide adenine dinucleotide cofactors NAD(P)H.1h, 2 In addition to these, a range of alternative regeneration approaches have been developed.^3^ For example, chemical reductants ranging from synthetic nicotinamide mimics^4^ to transition metal reductants,^5^ as well as electrochemical^6^ of photochemical^7^ regeneration methods have been reported.

Elemental metals represent another, potentially useful source of reducing equivalents to drive OYE‐catalyzed reduction reactions. To the best of our knowledge, apart from some pioneering works by Schwaneberg et al.,^8^ this approach has not been explored further in redox biocatalysis.

We therefore decided to explore the potential of elemental metals to promote OYE‐catalyzed reduction reactions (Scheme 1). Principally, two mechanisms can be imagined for the electron transfer from the metal to the enzyme‐bound flavin prosthetic group. Either direct, sequential single‐electron transfer occurs from the metal surface to the enzyme active site (the prosthetic flavin cofactor, respectively) or, as the prosthetic group is usually buried deeply within the protein, indirect electron transfer via a low‐molecular weight mediators is established.

**Scheme 1:**
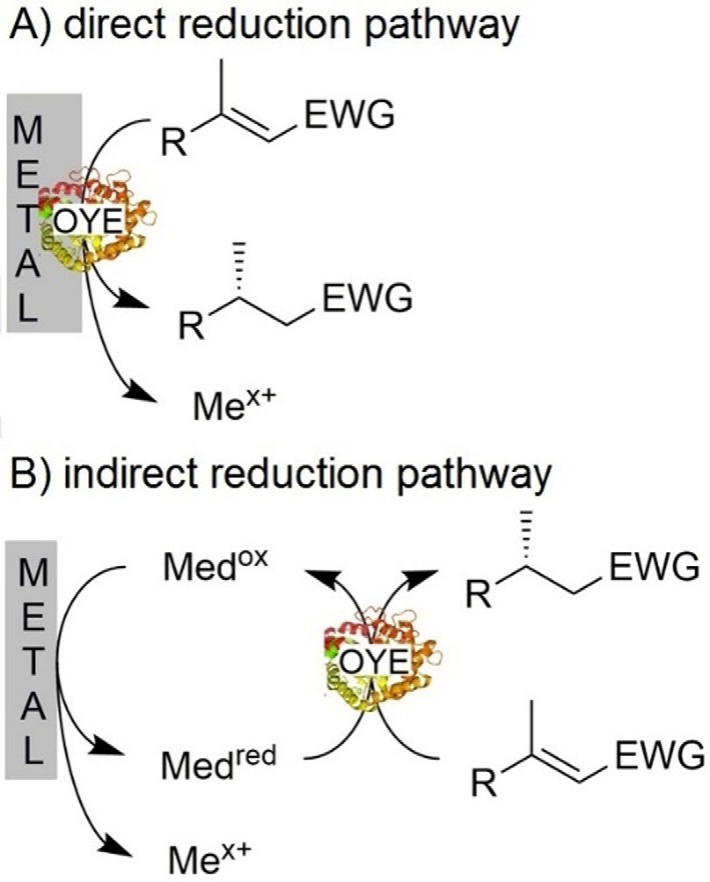
Metal‐driven regeneration of OYE via either A) direct electron transfer to the enzyme‐bound prosthetic group or B) indirectly using a low‐molecular‐weight mediator.

As a model enzyme we chose the OYE homologue from *Bacillus subtilis* (YqjM).2c, 9 For this, we evaluated pathway A, that is, direct electron transfer by incubating purified YqjM in the presence of a metal and recording UV spectra of the reaction mixture over time. Particularly, elemental zinc and chromium gave positive results (Figure 1). The disappearance of the characteristic peak around 450 nm for the oxidized flavin prosthetic group served as analytical signal to detect electron transfer.

**Figure 1:**
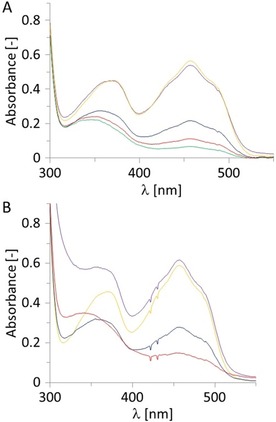
Direct reduction of YqjM using A) Zn or B) Cr as reductant. Conditions: [purified YqjM]=50 μm in KPi buffer 100 mm pH 6.5, 65 mg mL^−1^ of zinc powder <10 μm or 50 mg mL^−1^ of chromium powder <45 μm, 21 °C. Spectra were recorded at intervals A) 0 (yellow), 10 (blue), 20 (red), 30 min (green) and after re‐oxidation (purple); B) 0 h (yellow), 6 (blue), 23 h (red) and after re‐oxidation (purple, note that the FMN spectrum is overlaid by the presence of chromium ions leached into the reaction medium).

Interestingly, whereas YqjM was fully reduced within minutes in the presence of Zn, (Figure 1 A), it took several hours to fully reduce YqjM with Cr (Figure 1 B). The exact reason for this is unclear. Interestingly, the standard redox potential does not seem to have a decisive influence (*E*°(Zn/Zn^2+^)=−0.76 V_NHE_, *E*°(Cr/Cr^2+^)=−0.9 V_NHE_). It is worth mentioning that upon aeration the spectrum of the oxidized flavin prosthetic group was fully restored indicating that the reduction by the metals had no detrimental effect on the enzyme integrity.

However, performing chemoenzymatic reductions of 2‐methylcyclohexenone with Zn as sacrificial electron donor yielded only very poor optical purities of the products (Figure S3 in the Supporting Information). We attributed this to a significant nonenzymatic background reduction yielding racemic products, which we also confirmed in the corresponding control reactions (Figure S4). Therefore, for all further reactions, we used Cr as sacrificial electron donor. Similar to YqjM, further OYEs such as the *Sc*OYE2 from *Saccharomyces cerevisiae* and *Ts*OYE from *Thermus scotoductus* were also reduced directly by Cr and catalytically reduced 2‐methylcyclohexenone (Table S3).

In view of the rather sluggish reduction kinetics using Cr metal alone (Figure 1), we investigated several mediators (Scheme 1 B). As shown in Figure 2, all redox active mediators tried significantly improved the product formation approximately tenfold compared with the direct, nonmediated reduction.

**Figure 2:**
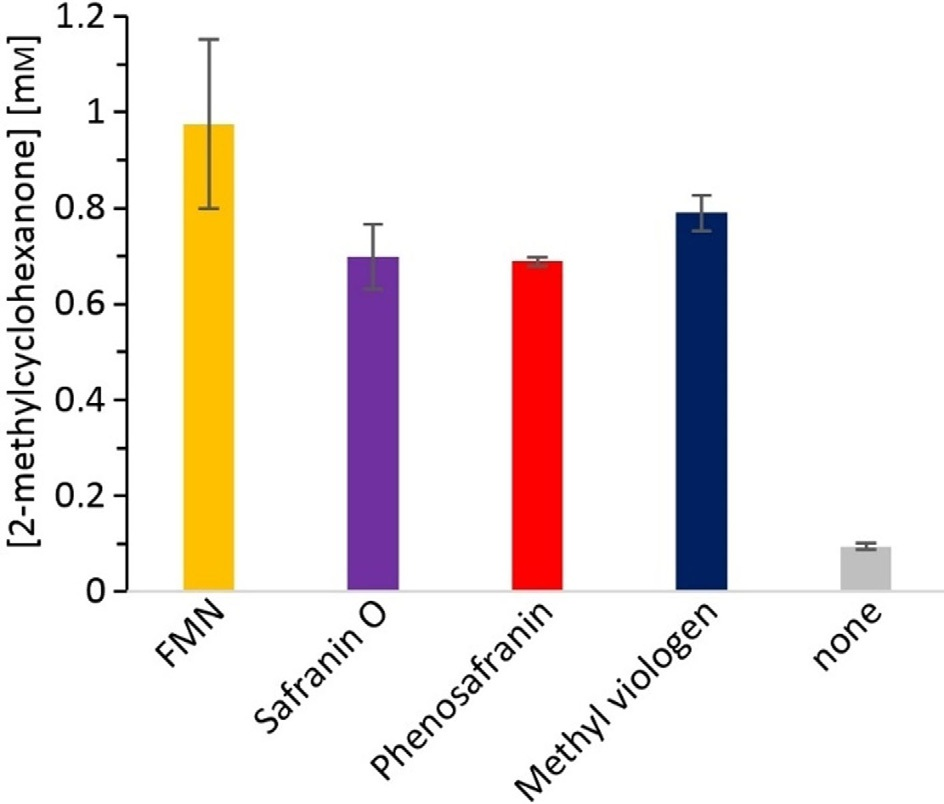
Chemoenzymatic reduction of 2‐methylcyclohexenone in the presence/absence of low‐molecular weight redox mediators. Conditions: [purified YqjM]=6.9 μm, [2‐methylcyclohex‐2‐en‐1‐one]=10 mm, 50 mg mL^−1^ of chromium powder <45 μm, [mediator]=500 μm in KPi buffer 100 mm pH 6.5, 21 °C (room temperature), reaction time: 4 h. Data presented are an average of duplicates (±SD).

We therefore performed all further experiments using FMN as mediator. In a next step, we investigated the influence of the amount of the reductant (Cr) on the overall performance of the reduction reaction. As shown in Figure 3, an almost linear relationship between amount of Cr and the final product concentration was observed. It must, however, be noted that a 1000‐fold molar excess of Cr over the starting material was necessary to attain full conversion. A plausible explanation for this observation may be that passivation of the metal surface prevented the bulk metal from being used for the reduction of FMN. Additionally, the Cr metal used in this study was already partially passivated as XPS analysis revealed a Cr^o^‐surface content of approximately 5.5 % (after a reaction this value was reduced to 1.2 %, see Section 6.5 in the Supporting Information).

**Figure 3:**
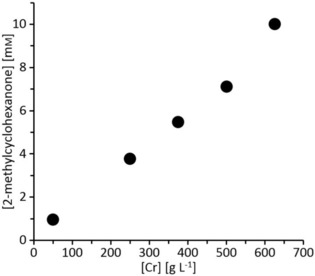
Influence of the Cr amount used on the overall product formation. Conditions: [purified YqjM]=6.9 μm, [FMN]=500 μm in KPi buffer 100 mm pH 6.5, [2‐methylcyclohex‐2‐en‐1‐one]=10 mm, chromium powder <45 μm, 21 °C reaction time: <4 h, please note: in case of low Cr loadings maximal conversion was observed already after several minutes. Data presented are averages of duplicates.

The product scope of the proposed chemoenzymatic reaction was very similar to the scope determined previously.2b Also the enantioselectivities were comparable, with the exception of levodione and citronellal, for which the enantiomeric excess (*ee*) values of the chemoenzymatic reaction system were significantly lower than those obtained using NAD(P)H as reductant. Control experiments in the absence of YqjM revealed a significant background activity (formation of racemic reduction product) which can be attributed to the direct, nonenzymatic reduction by the reduced mediator (Tables 1 and S4). Similar observations have been reported by Scrutton and co‐workers.7b Interestingly, no such side reaction was observed using Cr alone as reductant.

**Table 1:** Substrate scope of the chemoenzymatic reduction.^[a]^

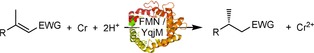				
Product	Conc.	Conversion	Yield	*ee*
	[mm]	[%]^[b]^	[%]^[c]^	[%]
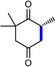	8.6	81	81.1	65 (*R*)
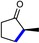	6.9	70	69.6	85 (*S*)
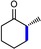	7.3	70	70.1	>99 (*R*)
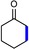	6.7	77	77.0	–
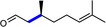	2.8	26	26.1	n.d.^[d]^
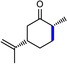	0.6	85	6.0	>99 (2*R*,5*S*)

[a] Conditions: [purified YqjM]=6.9 μm, [FMN]=500 μm in KPi buffer 100 mm pH 6.5, [substrate]=10 mm, 500 mg mL^−1^ of chromium powder <45 μm, room temperature, reaction time: 4 h. [b] Conversion=([starting material]_0_−[starting material]_4h_)×[starting material]_0_^−1^. [c] Yield=[product]_4h_×Σ[all reagents]_4h_^−1^. [d] Not determined.

The enantioselectivities observed in our reaction system compared well with those obtained in previous studies using traditional regeneration schemes.2b The evaluation of the substrate scope (Table 1) also revealed the generally very high chemoselectivity of the proposed reaction scheme. For the reduction of carvone, control experiments showed that the poor chemoselectivity here can be attributed to the direct, nonenzymatic reduction of the nonconjugated C=C double bond by the reduced mediator (Figure S5). Once more, no such side reaction was observed using Cr alone as reductant.

Traditional, that is, NAD(P)H‐dependent, regeneration systems are challenged by poor selectivity as well. Especially when using crude (i.e., nonpurified) enzyme preparations, over‐reduction of the carbonyl group is frequently observed, in particular with conjugated aldehydes as starting materials.^10^ As the reaction system proposed here does not regenerate reduced nicotinamide cofactors, this undesired side reaction should play no role here. We therefore also tested crude cell extracts of recombinant *Escherichia coli* cells overexpressing YqjM as biocatalyst preparation for the reduction of 2‐methylcyclohex‐2‐en‐1‐one and citral. In both cases, the saturated ketone and aldehydes were observed as sole products without formation of the undesired alcohol by‐products (Table S5).

Overall, in this contribution we have demonstrated that elemental metals can serve as sacrificial reductants to promote OYE‐catalyzed C=C bond reductions. Though the direct, unmediated electron transfer to the enzymes was feasible, it was greatly accelerated by the application of redox mediators. The high chemoselectivity of nicotinamide‐independent regeneration systems was preserved. This approach might particularly prove useful for substrate screening campaigns.

The current system, apparently, needs significant further improvements to become truly practical. First and foremost, using Cr as stoichiometric reductant does not appear to be a truly green method, especially considering the need for high molar surpluses and the release of questionable metal ions into the reaction medium. We envision that mixed valence state oxides or sulfides may be more practical alternatives to elemental metals as here the release of metal ions into the reaction medium may be avoided. Also, the particles can principally be recharged electrochemically and re‐used.

## Experimental Section

**Enzyme preparation**: Recombinant expression and purification of YqjM from *B. subtilis* was performed following a previously described procedure.9c

**Chemoenzymatic reactions**: Each reaction was carried out under strict anaerobic conditions (glovebox) in 1.5 mL glass vials with a working volume of 400 μL. Stirring bars were used for suspending the metals in the reaction. Reactions with mediators were protected from light with aluminum foil.

**XPS analysis**: The used XPS was a Thermo Fisher K‐Alpha. The X‐ray gun uses an AI_Kα_ source with an energy of 1486 eV. The (nominal) spot size was set to 400 μm. During the measurements a flood gun was used for charge compensation, setting the pressure to approximately 5.10^−7^ mbar.

## Conflict of interest

*The authors declare no conflict of interest*.

## Supporting information

As a service to our authors and readers, this journal provides supporting information supplied by the authors. Such materials are peer reviewed and may be re‐organized for online delivery, but are not copy‐edited or typeset. Technical support issues arising from supporting information (other than missing files) should be addressed to the authors.

Supplementary
