# Supplementary material for: Metals in Biotechnology: Cr‐Driven Stereoselective Reduction of Conjugated C=C Double Bonds
Source: Chembiochem. 2019 Dec 19;21(8):1112–5. doi: 10.1002/cbic.201900685 (PMC7217005; doi:10.1002/cbic.201900685)
Supplement: Supplementary file 1 — Supplementary [file CBIC-21-1112-s001.pdf]

CHEMBIOCHEM

Supporting Information

Metals in Biotechnology: Cr-Driven Stereoselective Reduction of Conjugated C=C Double Bonds

Marine C. R. Rauch+,[a] Yann Gallou+,[a] Léna Delorme,[a] Caroline E. Paul,[a] Isabel W. C. E. Arends,[b] and Frank Hollmann*[a]

cbic_201900685_sm_miscellaneous_information.pdf

1. General information

Unless stated otherwise all chemicals were purchased from Sigma-Aldrich (Steinheim, Germany), New England Biolabs (Ipswich, MA, USA) or Merck (Darmstadt, Germany) in the highest quality available and used without further purification.

2. Expression and purification of YqjM

E. coli BL21 (DE3) cells harboring pET28aHis-YqjM[1] were used for the expression of YqjM. Expression of the YqjM gene was carried out by inoculating 1 L of autoinduction ZYM-5052 media[2] supplied with 50 µg/mL kanamycin with 100 mL of overnight culture. Cells were grown overnight at 37 °C in baffled shake flasks. Cells were harvested by centrifugation (10000 × g, 15 min, 4 °C), washed with potassium phosphate buffer (20 mM, pH 6.5) and centrifuged again at the same speed. Subsequently, the cell pellet was resuspended in the same buffer. Mechanical cell disruption was effected by using Multi Shot Cell Disruption System (Constant Systems Ltd, Daventry, UK). Cell debris was separated from the crude extract by centrifugation at 10000 × g for 30 min at 4 °C. From there, one part was kept as cell-free extract and the rest was purified. The rest of supernatant was loaded to 14 mL Ni-NTA chromatography column (Thermo Fisher Scientific Inc.). The loading was performed using NGCTM Chromatography system (Bio-Rad). After loading, various successive washing with 20 mM potassium phosphate 30 mM imidazole pH 6.5 were performed in order to eliminate other proteins. Then, the elution of YqjM was performed by using elution buffer (20 mM potassium phosphate 250 mM imidazole pH 6.5) in a gradient 0-100% during 7 column volumes. Fractions containing proteins were tested with UV assay (reduction of 2-cyclohexen-1-one) and those containing YqjM were collected and incubated with 5 mM FMN. After 30 min of incubation on ice, enzyme suspension was desalted twice using PD-10 Desalting Columns (GE Healthcare) in order to remove the excess of FMN, and concentrated using Amicon® Ultra-15 Centrifugal Filter Device (cut-off 30 kDa).

3. Determination of YqjM activity

The YqjM activity assay was established with 2-methylcyclohex-2-en-1-one as standard substrate. The consumption of NADPH during the enzymatic reaction was directly followed at 340 nm for 120 s. The 2-methylcyclohex-2-en-1-one concentration in the assay was 1 mM and the initial concentration of NADPH was 150 µM. Because of background activity with oxygen, glucose and glucose oxidase was also added at a concentration of 20 mM and 10 U/mL respectively. The buffer used was KPi buffer 100 mM pH 6.5.

4. Biocatalytic reduction of 2-methylcyclohex-2-en-1-one by YqjM regenerated by metals

Each reaction was carried out in the glovebox in a 1.5 mL glass vial with a working volume of 400 µL. To ensure the absence of oxygen in the reaction, each component was placed in the glovebox at least few hours before use. Stirring bars were used for getting a good suspension of metals in the reaction. Reactions with mediators were protected from the light with an aluminium foil. Duplicates were performed.

50 mg/mL of chromium (or 65 mg/mL of zinc) correspond to a concentration of 1 M in the reaction.

For analysis, as each time-point corresponds to one reaction glass vial, 400 µL of ethyl acetate (containing 5 mM of dodecane as internal standard) was added for performing the extraction. The separation of the two phases was obtained *via* centrifugation. The combined organic phases were dried over anhydrous $MgSO_4$ and transferred into a GC vials for analysis.
All concentrations reported on this paper are based on calibration curves obtained from authentic standards and treated in the same manner as described here. For 2-methylcyclohex-2-en-1-one, the *ee* value during this study was >90%, knowing that the substrate is only pure at 90% (the 10% of impurity is a racemic mixture of 6-methylcyclohex-2-en-1-one which can also be reduced by YqjM).

5. Analytical procedures

5.1. Gas Chromatography (GC)

GC measurements were performed on Shimadzu GC-14A/FID or Shimadzu GC-2010 plus/FID equipped with different columns (Table S1).

Table S1: GC analytics

Substrate/product	column	temperature program/ gradient	retention time
2-methylcyclohex-2-en-1-one / (*S*)- or (*R*)-2-methylcyclohexan-1-one	GC<br>CP-Chirasil-Dex-CB (Agilent)<br>(25 m × 0.32 mm × 0.25 µm)<br>carrier gas: He	70°C hold 2 min<br>2°C/min to 80°C hold 2.0 min<br>2°C/min to 90°C hold 3.0 min<br>25°C/min to 150°C hold 1.0 min<br>25°C/min to 225°C hold 1.0 min	16.8 min (*S*)-2-methylcyclohexan-1-one<br>17.9 min (*R*)-2-methylcyclohexan-1-one<br>18.1 min 2-methylcyclohex-2-en-1-one<br>18.7 min / 18.8 min 2-methylcyclohexenol<br>20.2 min dodecane (IS)
Citral/ citronellal	GC<br>CP-Chirasil-Dex-CB (Agilent)<br>(25 m × 0.32 mm × 0.25 µm)<br>carrier gas: He	80°C hold 1 min<br>4°C/min to 85°C hold 5.0 min<br>4°C/min to 90°C hold 15.0 min<br>4°C/min to 100°C hold 15.0 min<br>4°C/min to 120°C hold 5.0 min<br>25°C/min to 225°C hold 1.0 min	22.7 min citronellal<br>25.4 min octanol (IS)<br>35.9 min / 41.9 min citral
2-methylcyclopenten-1-one / 2-methylcyclopentanone	GC<br>CP-Chirasil-Dex-CB (Agilent)<br>(25 m × 0.32 mm × 0.25 µm)<br>carrier gas: He	60°C hold 25 min<br>15°C/min to 65°C hold 4.0 min<br>15°C/min to 225°C hold 1.0 min	12.9 min (*S*)-2-methylcyclopentanone<br>13.1 min (*R*)-2-methylcyclopentanone<br>16.8 min 2-methylcyclopentenone<br>34.6 min dodecane (IS)
Ketoisophorone / levodione	GC<br>CP-Chirasil-Dex-CB (Agilent)<br>(25 m × 0.32 mm × 0.25 µm)<br>carrier gas: He	110°C hold 4.0 min<br>5°C/min to 130°C hold 5.0 min<br>20°C/min to 220°C hold 1.0 min	6.9 min octanol (IS)<br>9.1 min ketoisophorone<br>10.0 min (*R*)-levodione<br>10.4 min (*S*)-levodione
(+)-carvone / (+)-dihydrocarvone	GC<br>CP-Chirasil-Dex-CB (Agilent)<br>(25 m × 0.32 mm × 0.25 µm)<br>carrier gas: He	80°C hold 5.0 min<br>10°C/min to 90°C hold 10.0 min<br>10°C/min to 100°C hold 10.0 min<br>25°C/min to 235°C hold 1.0 min	20.4 min dodecane (IS)<br>28.4 min (2*S*,5*S*)-(+)-dihydrocarvone<br>28.5 min (2*R*,5*S*)-(+)-dihydrocarvone<br>28.9 min 5-isopropyl-2-methylcyclohex-2-en-1-one<br>29.5 min (+)-carvone
2-cyclohexenone / 2-cyclohexanone	GC<br>CP-wax-52-CB (Agilent)<br>(50 m × 0.53 mm × 2 µm)<br>carrier gas: N_2	70°C hold 8.0 min<br>25°C/min to 120°C hold 6.0 min<br>25°C/min to 250°C hold 1.0 min	7.1 min dodecane (IS)<br>10.0 min 2-cyclohexanone<br>12.5 min 2-cyclohexenone

5.2. Spectrophotometer

The instrument used is a Cary 60 UV-Vis spectrophotometer (equipped with a single cell Peltier accessory) from Agilent technologies.

5.3. XPS analysis

The used XPS was a ThermoFisher K-Alpha. The X-ray gun uses an Al Kα source with an energy of 1486 eV. The (nominal) spot size was set to 400 µm. During the measurements a flood gun was used for charge compensation, setting the pressure to about 5.10^{-7} mbar.

6. Supporting data

6.1. Stability assay of YqjM toward chromium

Stability assays of YqjM in presence of chromium species have been performed. Table S2 demonstrate that chromium species do not have an influence on YqjM's residual activity after 24 hours.

Table S2. Residual activity of YqjM in presence of different chromium species after 24 hours. Conditions: [YqjM] = 6.9 µM in KPi buffer 100 mM pH 6.5, $[Cr]_{powder}$ = 50 mg/mL or $[CrCl_2]$ = 1 mg/mL or $[CrCl_3]$ = 1 mg/mL. Data presented are an average of duplicates.

Chromium species	Residual activity after 24 hours
Chromium powder	97%
Chromium (II) chloride	80%
Chromium (III) chloride	84%

6.2. Regeneration of further OYE by Cr

Table S3. Enzyme scope of the regeneration of OYEs by chromium. Conditions: [purified enzyme] = 6.9 µM in KPi buffer 100 mM pH 6.5, [2-methylcyclohex-2-en-1-one] = 10 mM, 50 mg/mL of chromium powder <45 µm, 21 °C. Data presented are an average of duplicates.

Enzyme	Product formation (mM)	TF (h^{-1})
YqjM	0.40	0.80
*Sc*OYE2	0.51	1.45
*Ts*OYE	0.16	0.45

6.3. Control reactions of substrate in presence of chromium and FMN

Control reactions have been performed for determining the chemical background of the system. When only chromium was in presence of substrates, no reduction was observed. However when FMN and chromium were present, ketoisophorone and citral were reduced in a racemic mixture.

Table S4. Control reactions of substrate in presence of chromium and FMN. Conditions: [FMN] = 500 µM in KPi buffer 100 mM pH 6.5, [substrate] = 10 mM, 500 mg/mL of chromium powder <45 µm, 21 °C, reaction time of 4 hours. Data presented are an average of duplicates.

Substrate	Racemic product formation (mM)
ketoisophorone	6.8
citral	1.8

6.4. Data on experiments with CFE

Table S5. Characterisation of the use of CFE YqjM. [CFE YqjM] = 6.9 µM, [FMN] = 500 µM in KPi buffer 100 mM pH 6.5, [substrate] = 10 mM, 500 mg/mL of chromium powder <45 µm, 21 °C, reaction time of [a] 4 hours or [b] 20 hours. Data presented are an average of duplicates.

Substrate	Expected product	Product concentration (mM)	Yield (%)	*ee* (%)
2-methyl-2-cyclohexenone 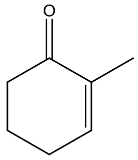	2-methylcyclohexanone 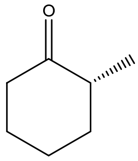	4.4 [a]<br>6.0 [b]	42 [a]<br>60 [b]	93 [a]<br>81[b]
Citral 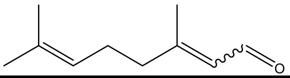	Citronellal 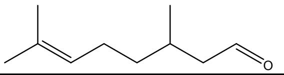	1.5 [a]<br>3.9 [b]	16 [a]<br>38 [b]	n.d.

Yield = [expected product] / $[substrate]_0$
n.d. = not determined.

6.5. XPS analysis

XPS measurements have been performed on a sample of chromium powder (sample 1) and on a sample of powder chromium used for the regeneration of YqjM (Sample 2). On each samples, two measurements have been performed and these duplicates are reproducible. In Table S5, the content of each sample is summarised.

Table S6. Summary of the higher elements present in the samples with their peak binding energy and atomic%.

	Chromium powder (Sample 1)		Chromium powder used (Sample 2)	
	Peak Binding energy	Atomic %	Peak Binding energy	Atomic %
O1s	529.89	45.48	530.90	41.49
C1s	284.45	26.51	284.45	35.54
Cr2p	575.28	16.69	575.63	3.54
Fe2p	710.56	6.04	710.88	4.29
Si2p	101.12	1.93	102.51	2.22
N1s	396.30	1.41	399.43	3.85
P2p	-	-	132.95	6.33
K2p	-	-	292.41	0.47

Phosphate and potassium are present in the sample 2 due to the use of KPi buffer for the reaction. Sample 2 has been washed but small quantities of phosphate and potassium are still present at the surface of the particles.

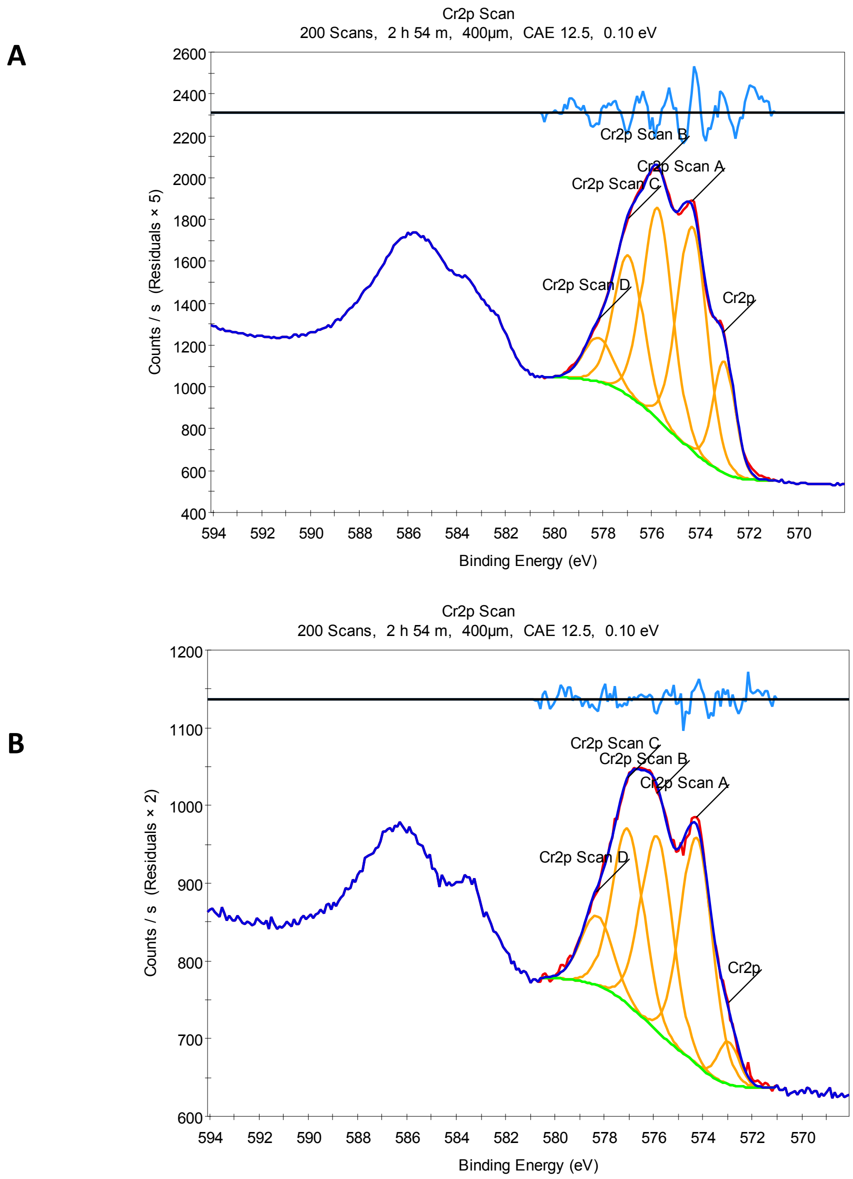

Figure S1. XPS spectra of Cr of the sample 1 (A) and sample 2 (B).

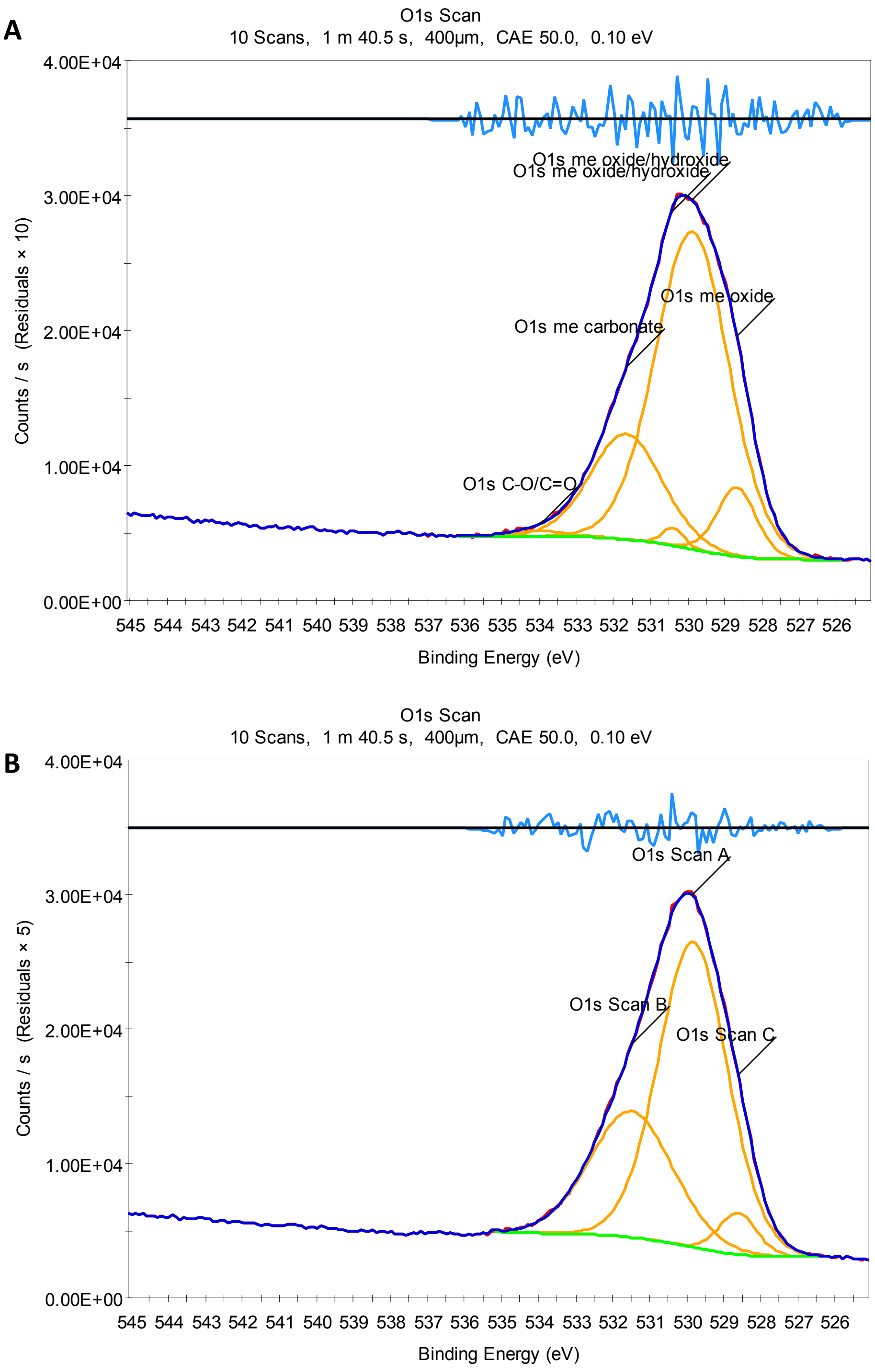

Figure S2. XPS spectra of O of the sample 1 (A) and sample 2 (B).

6.6. GC chromatograms of the chemical reduction of 2-methylcyclohexenone by zinc

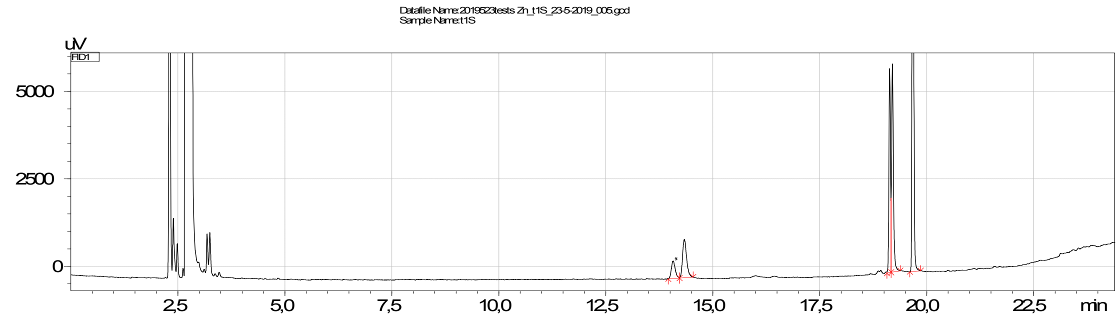

Figure S3. Chromatogram of the chemoenzymatic reduction of C=C-double bonds of 2-methylcyclohexenone by YqjM regenerated with zinc. The reaction is not enantioselective and 2-methylcyclohexenol is also produced.

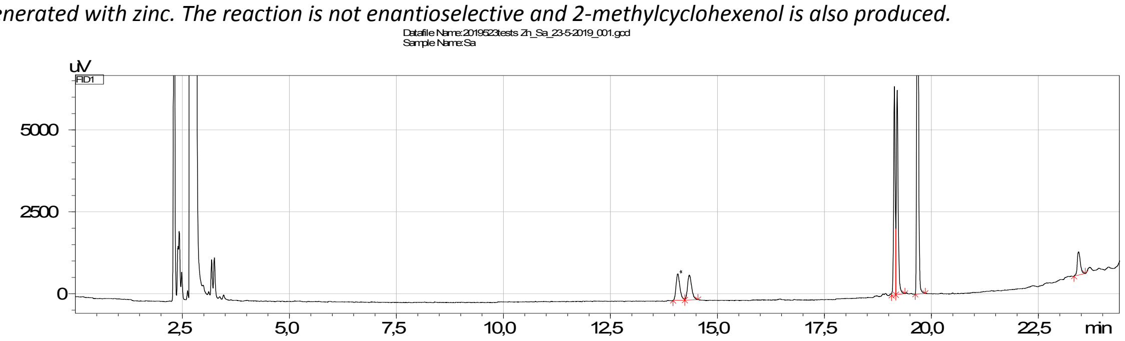

Figure S4. Chromatogram of the chemical reduction of 2-methylcyclohexenone by zinc. Zinc reduced the C=C-double bond and the ketone.

6.7. GC chromatograms of the chemical reduction of carvone by reduced mediators

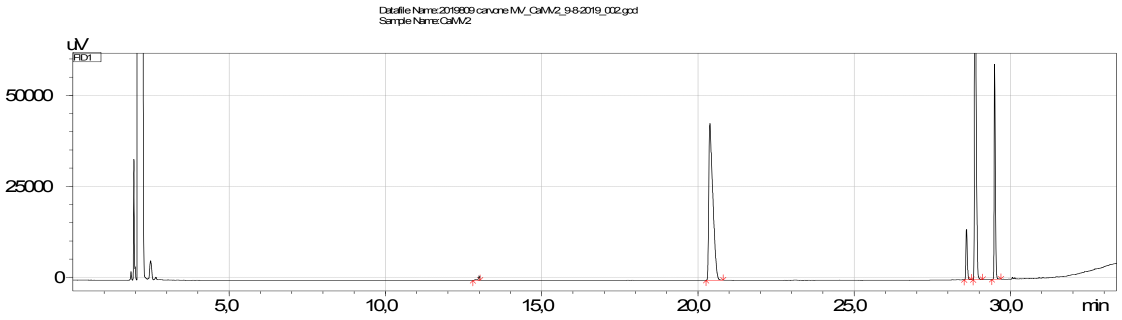

Figure S5. Chromatogram of the chemical reduction of carvone by reduced mediators. The peak at 28.9 min corresponds to the reduction of the non-conjugated C=C-double bond.

7. References

1. T. Classen, M. Korpak, M. Scholzel, J. Pietruszka, *ACS Catal.*, **2014**, *4*, 1321-1331.
2. F. W. Studier, *Methods Mol. Biol.*, **2014**, *1091*, 17-32.
